# Pregenual Anterior Cingulate Gyrus Involvement in Spontaneous Social Interactions in Primates—Evidence from Behavioral, Pharmacological, Neuropsychiatric, and Neurophysiological Findings

**DOI:** 10.3389/fnins.2017.00034

**Published:** 2017-02-01

**Authors:** Can Van Mao, Mariana F. P. Araujo, Hiroshi Nishimaru, Jumpei Matsumoto, Ahn Hai Tran, Etsuro Hori, Taketoshi Ono, Hisao Nishijo

**Affiliations:** ^1^System Emotional Science, Graduate School of Medicine, University of ToyamaToyama, Japan; ^2^Edmond and Lily Safra International Institute of Neuroscience, Santos Dumont InstituteMacaiba, Brazil

**Keywords:** social interactions, freely behaving monkeys, social cognition, anterior cingulate cortex, single neuron activity

## Abstract

The anterior cingulate cortex (ACC) has been implicated in different aspects of cognition and decision making, including social cognition. Several studies suggest that this region is actually formed by sub-regions concerned with distinct cognitive functions. The ACC is usually divided in its rostro-caudal axis, with the caudal ACC playing a major role in processing own actions, and the rostral ACC being related to social cognition. Recently, it has been suggested that the ACC can also be functionally divided in its dorso-ventral axis into ACC gyrus (ACCg) and ACC sulcus (ACCs), with the ACCg having a central role in processing social information. In this context, we propose that the pregenual ACCg might be especially important for engaging in social interactions. We discuss previous findings that support this hypothesis and present evidence suggesting that the activity of pregenual ACCg neurons is modulated during spontaneous social interactions.

## Introduction: a role of the ACC in social behaviors in primates

The anterior cingulate cortex (ACC) is one of the pivotal components in the brain network. It has been implicated in different aspects of cognition and decision-making, such as in working memory, anticipation, response selection (Procyk et al., 2000; Koyama et al., 2001; Hoshi et al., 2005), error detection and reward prediction (Amiez et al., 2005; Shidara et al., 2005; Matsumoto et al., 2007), conflict monitoring (Botvinick et al., 2004), and behavioral shift after error (Kawai et al., 2015). This region is also involved in emotion processing and social cognition (Bush et al., 2000; Amodio and Frith, 2006; Rushworth et al., 2007). Neuroimaging studies, for example, have indicated that the medial prefrontal cortex, including the ACC, is involved in mental state attributions (Ciaramidaro et al., 2007; Steinbeis and Koelch, 2009). In addition, postmortem and clinicopathological studies have reported morphological changes in the ACC in patients with schizophrenia and bipolar disorder (Wang et al., 2007; Calabrese et al., 2008; Meisenzahl et al., 2008; Bersani et al., 2014), while functional magnetic resonance imaging studies have linked functional deficits in the ACC with schizophrenia (Britton et al., 2006; Borgwardt et al., 2008), especially its negative symptoms (Bersani et al., 2014; Nelson et al., 2015).

The results of many studies in primates have also suggested a role of the ACC in social cognition. A previous study reported that ACC functional connectivity was linked to social network size; the functional coupling between the ACC and the superior temporal sulcus, another region of the brain that is related to social cognition, increased with social group size (Sallet et al., 2011). Such an increase may be related to the need to predict the behavior of more cage mates in order to adjust their own behavior (Rushworth et al., 2013). Accordingly, the ACC seems to play a role in social-based decision-making. Recent studies have suggested that the ACC processes information about not only self-generated actions and the related outcomes, but also observed actions and outcomes (Araujo et al., 2012). In addition, the activity of a group of ACC cells is able to predict the decisions of others, an essential ability for successful cooperative interactions (Haroush and Williams, 2015). Furthermore, the activity of this region of the brain has been shown to be related to reward allocations to self, others, or both (Chang et al., 2013). Consistent with the findings of all of these studies, lesions of the ACC induce deficits in social behavior in monkeys (Hadland et al., 2003; Rudebeck et al., 2006).

## ACC role in social behaviors: pharmacological and neuropsychiatric evidence

Schizophrenic patients display various behavioral impairments. Disturbances in social skills (e.g., avoiding social contact, neglecting a surrounding environment, social isolation) are the most pervasive aspects of schizophrenic patients. There are two widely accepted neurochemical hypotheses of schizophrenia, the dopamine hypothesis and the N-methyl-D-aspartate (NMDA) hypothesis. These hypotheses are based on observation that phencyclidine (PCP), a non-competitive NMDA-type glutamate receptor antagonist, and methamphetamine (MAP) or amphetamine, agents increasing dopamine release, could produce a variety of symptoms similar to human schizophrenic symptoms (Bell, 1965; Snyder, 1973; Ridley et al., 1982; Javitt and Zukin, 1991; Adler et al., 1999; Tsapakis and Travis, 2002). In animals, both compounds PCP and MAP induced abnormal behaviors such as hyperactivity, increased locomotors activity, ataxia, rearing, stereotype, head weaving, withdrawal from social interaction, etc., which corresponded to certain aspects of schizophrenic symptoms (Miller, 1976; Scraggs and Ridley, 1979; Miczek and Yoshimura, 1982; Sams-Dodd, 1998; Castner and Goldman-Rakic, 1999; Linn et al., 1999; Balla et al., 2001). Especially, chronic intermittent low doses of PCP produced very similar metabolic and neurochemical changes in the rodent brain to those in schizophrenic patients with prefrontal dysfunctions (Morris et al., 2005). In monkeys, chronic low-dose PCP treatment also induced a significant decrease in all categories of the social behaviors, and the chronic PCP monkeys spent less time in proximity to other monkeys than the control monkeys (Mao et al., 2008). Acute MAP injection to the chronic PCP monkeys exacerbated behavioral effects of PCP (Mao et al., 2008). These results suggest that this primate model with chronic PCP and/or acute MAP induced symptoms similar to negative symptoms of schizophrenia (e.g., social isolation, blunt behaviors, and withdrawing social behaviors) (Ellenbroek and Cools, 2000; Marcotte et al., 2001; Tsapakis and Travis, 2002).

It has been reported that the ACC was most sensitive to acute administration of ketamine (NMDA blocker) in normal and schizophrenic subjects (Holcomb et al., 2001, 2005; Rowland et al., 2005). Furthermore, the ACC responses to ketamine were larger in schizophrenic patients than normal controls, and the changes in the ACC were correlated with schizophrenic scores (Holcomb et al., 2005). On the other hand, NMDA and AMPA receptor densities were increased in the ACC of schizophrenic patients (Zavitsanou et al., 2002). These results suggest that glutamatergic activity in the ACC is reduced in schizophrenia. In rodents, subchronic administration of PCP induced morphological changes in the ACC (Hajszan et al., 2006). Taken together, these findings suggest that chronic PCP administration might induce neuropathological changes in the monkey ACC similar to those in human schizophrenic patients, which consequently might change activity patterns of the ACC neurons related to social behaviors. These neuropathological changes in the ACC induced by chronic PCP administration might result in disturbance in social behaviors in monkeys.

Post-mortem and clinicopathological studies using individuals with schizophrenia also indicated deficits in the ACC, such as loss of gray matter volume, reduced neuronal, and glial density (Benes et al., 1986, 1991; Brown et al., 1986), reduction of neuronal soma size, and cluster of neurons (Chana et al., 2003), and decrease in the density of non-pyramidal neurons in layer II (Todtenkopf et al., 2005). Neuroimaging studies also showed morphological changes in the ACC of schizophrenic and bipolar disorder patients such as reduction of ACC volume (Takahashi et al., 2002; Wang et al., 2007; Calabrese et al., 2008), and reduced density of gray matter of the ACC (Meisenzahl et al., 2008). Recently, fMRI studies also revealed functional deficits in the ACC in schizophrenic patients (Fahim et al., 2004; Britton et al., 2006; Borgwardt et al., 2008). Moreover, evidence from psychiatric patients support the hypothesis that a specific part of the ACC, the pregenual ACC, has a central role in social cognition. In patients with schizophrenia with social deficits, the volume of the pregenual ACC is decreased (Suzuki et al., 2002). In patients with autism, resting state fMRI studies indicated that the functional connectivity of the pregenual ACC is decreased (Kennedy and Courchesne, 2008; Di Martino et al., 2009b) and that the activity of the pregenual ACC is decreased during a social task (Di Martino et al., 2009a). All of these pharmacological and neuropsychiatric evidence suggest a pivotal role of the ACC in social behaviors.

## Functional topography of the ACC: a role of the pregenual ACC gyrus in social cognition

The ACC was classically regarded as part of the limbic system. It is, however, formed by a number of different citoarchitectonic sub-regions, which suggests that different sub-regions may be involved in different functions (Vogt, 2009). Although there has been no absolute consensus on how the ACC is functionally divided, the ACC sub-regions are usually defined along its rostro-caudal axis. Several authors, based on findings from human functional magnetic resonance imaging and neurophysiological studies, have suggested that the ACC can be functionally divided into 2 subdivisions: the rostral part of the ACC activated by emotional tasks (the affective division), and the caudal part of the ACC activated by cognitive tasks (the cognitive division) (Bush et al., 2000; Davis et al., 2005; Kennerley et al., 2006). The results of rodent lesion and pharmacological studies have also supported this division (Johansen and Fields, 2004; Malin et al., 2007). Amodio and Frith (2006) have suggested that the ACC (together with other regions at the medial prefrontal cortex) is involved in determining behavior based on anticipated value. In this theoretical frame, caudal ACC would be involved in processing the value of actions, while rostral ACC would play a major role in many aspects of social cognition.

Recently, Apps et al. (2016) have suggested another division for the ACC. They have argued that the ACC gyrus (ACCg) is functionally distinct from the ACC sulcus (ACCs). According to their model, the ACCg would have a central role in processing social information.

In this context, we suggest that, among the rostro-caudal subdivisions of the ACC, the pregenual ACCg might be especially important for engaging in social interactions. This part of the ACC is connected with other emotion- and social cognition-related areas, such as the amygdala, insula, orbitofrontal cortex, and premotor area (Pandya et al., 1981; Amaral and Price, 1984; Beckmann et al., 2009; Morecraft et al., 2012).

## Pregenual ACCg neuronal activity during spontaneous social interactions in monkeys

So far, the neuronal basis, including the role of the pregenual ACC, of spontaneous social interactions is poorly understood. This is mainly due to the design of the majority of neurophysiological experiments in which the animals are usually restrained while they perform reward-based tasks. Thus, in order to investigate the activity of pregenual ACC neurons during social interactions in monkeys in more natural settings, we developed a social interaction paradigm in which 2 monkeys could spontaneously interact. The study was performed with 3 monkeys (2 *Macaca fuscata*, 1 *Macaca mulatta*; 2 females, 1 male) weighing 5–8 kg. Neuronal activity was recorded from the rostral ACC of 2 of the monkeys. The third animal was used as a partner in the social interaction task described below. All of the monkeys were treated in strict compliance with the United States Public Health Service Policy on Humane Care and Use of Laboratory Animals, the National Institutes of Health Guide for the Care and Use of Laboratory Animals, and the Guidelines for the Care and Use of Laboratory Animals at the University of Toyama. This study was approved by the Committee for Animal Experiments and Ethics at the University of Toyama. Every effort was made to minimize the number of animals used and their suffering.

The experimental sessions were conducted in 3 linked cages (300 cm long, 130 cm wide, and 160 cm high) that were separated by 2 mesh partitions. At the beginning of each session, 2 monkeys (1 recording monkey and a partner monkey) were put into the side cages. Then, the mesh partition of the side cage containing the recording monkey was removed, allowing the monkey to freely move inside the center cage and to get close to the partner monkey. Direct contact between the animals was prevented by the other mesh partition in order to avoid disconnection of the wiring, which is required for the neurophysiological recording, by the partner monkey. A camera with a charge-coupled device (CCD) was positioned on top of the cages, and it recorded the behavior of the animals. The video images were automatically analyzed online and stored for offline analysis. The behaviors of each monkey were detected and classified into one of the following categories: approach, leaving, self-grooming, moving around, proximity, contact, and communication (Table 1).

**Table 1:** **Categories and definition of monkeys' social behaviors analyzed in the neurophysiological experiment**.

**Behaviors of the recording monkey**	**Behaviors of the partner monkey**
**- Approaching 1:** The recording monkey approached to the partner monkey.	**- Approaching 2:** The partner monkey approached to the recording monkey.
**- Leaving 1:** The recording monkey left from the partner monkey.	**- Leaving 2:** Partner monkey left from the recording monkey.
**- Grooming 1:** The recording monkey groomed by themselves (self-grooming of the recording monkey).	**- Grooming 2:** The partner monkey groomed by themselves (self-grooming of the partner monkey).
**- Moving around 1:** The recording monkey moved around.	**- Moving around 2:** The partner monkey moved around.
**- Contact:** Both monkeys sat close together (distance between the 2 monkeys was less than 10 cm) without social behaviors.	
**- Proximity:** Distance between the 2 monkeys was 10–60 cm.	
**- Communication:** Both monkeys displayed a series of the social behaviors including grooming together, lip smacking, threatening, fighting, and moving around that occurred after they faced each other.	

Before the experiments started, a head-restraining device (a U-shaped resinoid plate) was surgically attached to the skull of the recording monkeys under aseptic conditions (Nishijo et al., 1988a,b; Tazumi et al., 2010). Each monkey was anesthetized with a combination of medetomizine hydrochloride (0.5 mg/kg, intramuscular injection) and ketamine hydrochloride (5 mg/kg, intramuscular injection). The plate was anchored with dental acrylic to tungsten bolts inserted in the skull.

One month after the surgery, when the monkeys were completely recovered from the surgery, head magnetic resonance imaging or X-ray scans were performed in order to locate the X-Y coordinates of the ACC. Then, the monkeys were trained to sit on a monkey chair with their head painlessly fixed to the stereotaxic apparatus through a head-restraining device. After training the monkeys to sit on the chair with their head painlessly fixed to a stereotaxic apparatus, the recording electrode assemblies were stereotaxically implanted above the ACC while the monkeys were under anesthesia. The recording assemblies were covered by a thin film of white petrolatum, and they were fixed with dental cement.

The recording electrode assemblies consisted of 4 electrodes, called tetrodes (tungsten wire, 20 μm in diameter; impedance, 200–400 kΩ 1 kHz), which were encased individually in a set of 4 stainless-steel guide tubes (33 gauge). The tubes were attached to a microdrive that consisted of a screw, which was coupled to a molded nut that was attached to the guide tubes (Sakurai and Takahashi, 2006; Ho et al., 2008).

Before each recording session, the heads of the monkeys were fixed painlessly onto the stereotaxic apparatus on the monkey chair. Then, the implanted tetrodes were lowered into the ACC with the microdrive while the neuronal activity was monitored on an oscilloscope. The tetrodes were lowered in 20-μm steps with a pause of 2 min between the steps. The maximum number of steps within 1 d was limited to 16 (i.e., 320 μm) in order to minimize damage to the brain.

The neuronal activity was passed through a high-input impedance preamplifier, amplified, and monitored on the oscilloscope. Only the neuronal activities with signal-to-noise ratios greater than 2.5–1.0 were used. When such neuronal activity was detected, the monkey was moved to the linked side cage. If the neuronal activity was still present for more than 30 min in the cage, it was judged stable and suitable for recording. The analog signals of the neuronal activities, the triggers for behavioral events that were emitted from the computer for the behavioral analysis, and the video signals from another CCD camera that was positioned on the side of the cages were digitized and stored in a computer through a Multichannel Acquisition Processor (Plexon Inc., Dallas TX, USA) system. The amplified neuronal signals were digitized at a 40-kHz sampling rate, and 1.0-ms waveforms that crossed an experimenter-defined threshold were stored on a computer hard disk for off-line spike sorting. The digitized neuronal activities were isolated into single units by their waveform components with the Offline Sorter program (Plexon Inc.). The Offline Sorter automatically concatenates (end-to-end) the waveforms of the 4 channels of the tetrode to make one quad-length waveform, and performs a principal component analysis based on the concatenated data points. Therefore, all of the principal components were calculated based on the total data that were derived from the tetrode. Each cluster was then checked manually to ensure that the cluster boundaries were well separated and that the waveform shapes were consistent with the action potentials. For each isolated cluster, an interspike interval histogram was constructed, and an absolute refractory period of at least 1.0 ms was used to exclude suspected multiple units. Finally, superimposed waveforms of the isolated units were drawn to check the consistency of the waveforms throughout the recording sessions, and they were then transferred to the NeuroExplorer program (Nex Technologies, Madison, AL, USA) for further analysis. Typically, 1–2 single units were isolated by means of an off-line cluster analysis from 4 channels (wires) of 1 tetrode.

The neuronal activities and social behaviors were recorded simultaneously. The computer that analyzed the data from the CCD camera emitted transistor-transistor logic (TTL) signals to the neuronal recording system when one of the behaviors occurred. Social behaviors were analyzed offline by visual inspection, and the timestamps for the behavioral events were added to the data manually.

**Figure 1:**
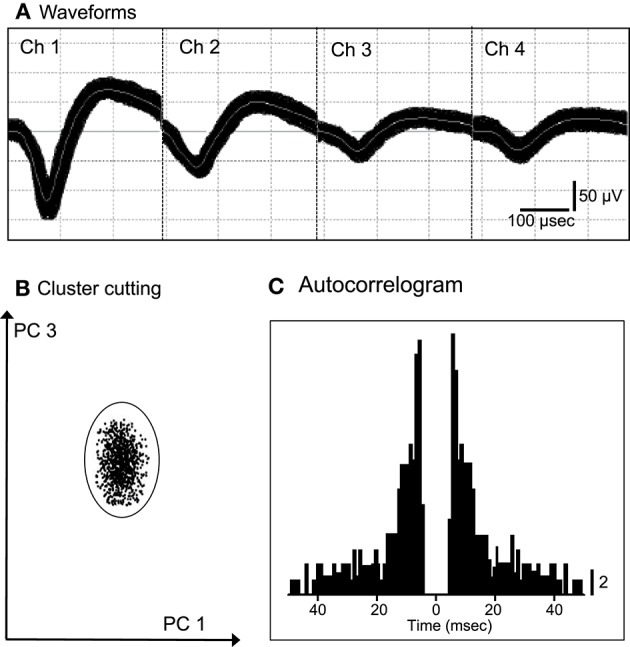
**An example of the raw records of a neuron in the anterior cingulate cortex (ACC). (A)** Superimposed waveforms recorded from 4 electrodes (tetrode). Chs. 1–4 indicate the signals from individual electrodes. **(B)** Results of the off-line cluster analysis. Each dot represents 1 neuronal spike. Only 1 cluster (circled) was recognized. **(C)** Autocorrelograms of the neurons indicated in **(A,B)**. Bin width, 1 ms. Calibration bar indicates the number of spikes per bin per trial.

We recorded 86 neurons from the ACC of the 2 monkeys. Figure 1 shows an example of the raw records of an ACC neuron. The typical waveforms of 1 ACC neuron that were simultaneously recorded from all channels of a tetrode (Chs. 1–4) are shown in Figure 1A. In contrast to the recordings from the rat hippocampus (Ho et al., 2008), usually 1 and only occasionally, 2 neurons per tetrode were encountered in the monkey ACC. Figure 1B shows the results of spike sorting by off-line cluster cutting of the neural activity shown in Figure 1A. Each dot represents 1 spike, and the cluster of dots that is encircled by the dotted lines was easily recognized. Figure 1C indicates an autocorrelogram of the neuron shown in Figure 1B. The autocorrelogram indicated that the refractory period of the neuron was 2–3 ms, indicating that these spikes were recorded from a single neuron.

**Figure 2:**
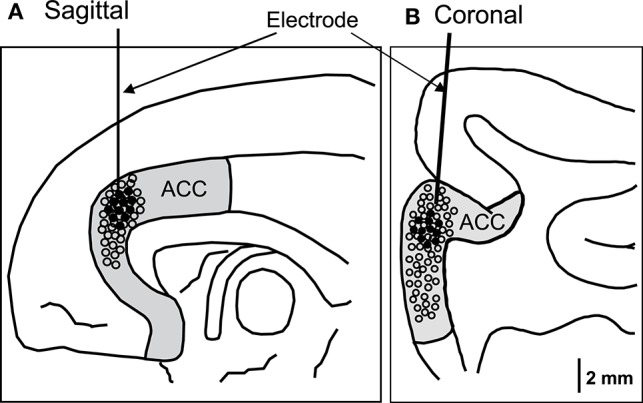
**Recording sites of the ACC neurons in the sagittal (A)** and coronal **(B)** views. The ACC neurons with activity that correlated with social behaviors were located in the rostral ACC. Filled circles, ACC neurons with significant responses; open circles, ACC neurons with insignificant responses.

For each recorded neuron, the mean neuronal firing rate 2 s before and 2 s after behavior onset was calculated and compared. Amongst them, 11 neurons responded to social behaviors, while none responded to non-social behaviors (self-grooming and moving around) (Wilcoxon signed rank test, *p* < 0.05). Figure 2 shows the recording sites of the ACC neurons. Most ACC-responsive neurons were recorded from the same sub-area within the ACC: the rostral ACC gyrus, located anterior to the genu of the corpus callosum.

### Characteristics of the responsive neurons

The responses of the neurons with activity related to social behavior felt into 3 categories: leaving-related (*n* = 6), leaving- and approaching-related (*n* = 1), or communication-related (*n* = 4) neurons. The leaving- and approaching-related activities changed in response to the leaving and approaching behaviors, respectively, of the recording and/or partner monkeys. The activity of the communication-related neurons changed when the 2 monkeys engaged in a series of mutual social behaviors, including grooming together, lip smacking, and facing.

**Figure 3:**
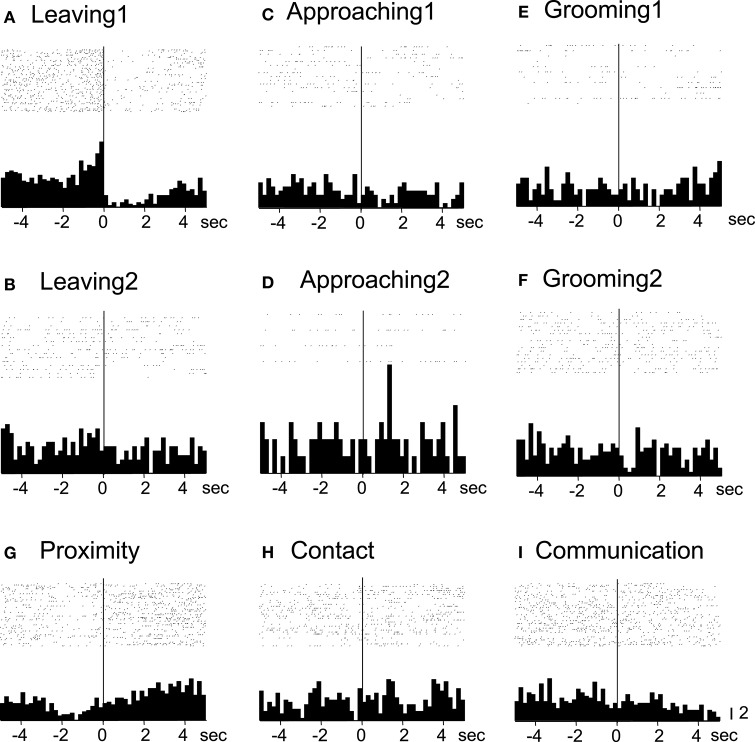
**Raster displays and summed peri-event histograms of the neuronal activity, which was correlated with the leaving behaviors of the recording monkey**. The activity of the neuron was inhibited in response to the leaving behaviors of the recording monkey (Leaving1, **A**), but not to the other behaviors **(B–I)**. Bin width, 100 ms.

**Figure 4:**
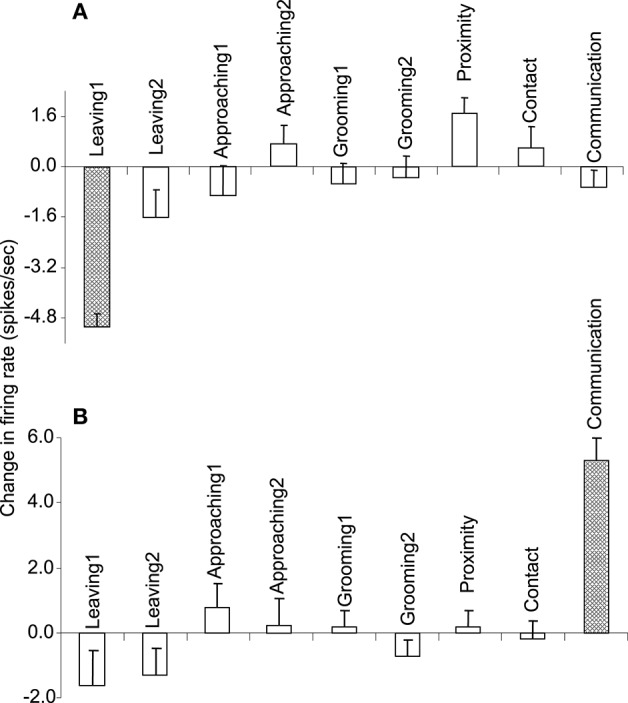
**The response magnitudes of the 2 neurons (A,B)** shown in Figures 3, and 5, respectively, that occurred in response to various social and non-social behaviors.

Of the 7 neurons with leaving-related activity, 4 responded when the recording monkey left the partner monkey (Leaving1) (3, excitatory; 1, inhibitory), and 3 responded when the partner monkey left the recording monkey (Leaving2) (all excitatory). The responses of a leaving-related neuron (Leaving1) are illustrated in Figure 3. The activity of the neuron was specifically inhibited in response to Leaving1 (Figure 3A) but not to Leaving2 (Figure 3B). However, the neuron responded neither to the approaching behaviors of the recording monkey (Approaching1, Figure 3C) nor to those of the partner monkey (Approaching2, Figure 3D). In addition, the neuron did not respond to the grooming behaviors of the recording monkey (Grooming1, Figure 3E) nor to those of the partner monkey (Grooming2, Figure 3F). Furthermore, the activity of the neuron did not change when both monkeys were located within a distance of 60 cm (Proximity, Figure 3G), when one of the monkeys touched the other monkey (Contact, Figure 3H), nor when both monkeys displayed a series of social behaviors (Communication, Figure 3I). The response magnitudes of the neuron to various behaviors are compared in Figure 4A.

**Figure 5:**
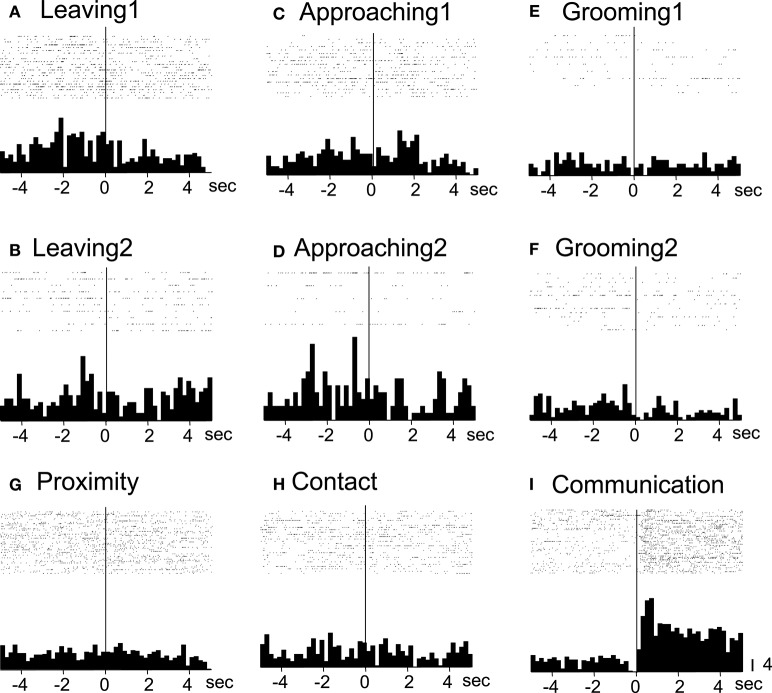
**Raster displays and summed peri-event histograms of the neuronal activity, which was correlated with communication**. The activity of the neuron was increased in response to communication of both of the monkeys (Communication, **I**) but not to the other behaviors **(A–H)**. Bin width, 100 ms.

All of the communication-related neurons displayed excitatory responses. Figure 5 illustrates an example of a communication-related neuron. The activity of the neuron increased specifically in response to communication (Figure 5I). The magnitudes of the responses of the neurons to various behaviors are summarized in Figure 4B. The activity of the neuron significantly increased in response to communication, in which both monkeys displayed a series of social behaviors, including grooming together, lip smacking, threatening, and fighting.

The activity of those ACC neurons cannot be ascribed to general locomotion because their activities were selective to approaching or leaving but not to both behaviors. In addition, the activity of none of those neurons was related to the behavioral category of moving around. Furthermore, direction of movement and social relations are important factors that characterize approaching and leaving behaviors; approaching and leaving behaviors involve moving toward and away from the other monkey, respectively. This selectivity to movement directions further support the idea that these neuronal activities were related to social behaviors and not to general locomotion. Another cingulate sub-region, the cingulate motor area (CMA), seems to be specialized in the cognitive control of voluntary motor behaviors (Dum and Strick, 1993; Walton and Marsm, 2007). The CMA neurons become active during various voluntary actions (Amiez et al., 2005; Hoshi et al., 2005) and also respond to external targets that are used for selecting an appropriate action (Isomura et al., 2003). These CMA neurons were located in the cingulate sulcus posterior to the rostral part of the ACC (Amiez et al., 2005; Hoshi et al., 2005). The present results, therefore, are in line with previous evidence suggesting a functional division of the ACC, with the most rostral areas being more closely related to social cognition (Amodio and Frith, 2006).

## The role of pregenual ACCg in social cognition

Electrophysiological studies in monkeys have also reported ACC neuronal activity during social interactions. Neurons located in posterior ACCs, for example, are activated during both self and observed actions and their related outcomes (Araujo et al., 2012), and ACCs activity can predict the others decision during social interactions (Haroush and Williams, 2015). Accumulating evidence, however, suggests that ACCs activity encodes information in a more self-centered perspective. Recently, Chang et al. (2013) recorded the activity of both ACCs and ACCg of monkeys while they performed a reward allocation task. They found that neurons in the ACCs encoded foregone reward (reward allocations to another monkey or to no one), suggesting that ACCs encodes rewards that are not allocated to oneself (Chang et al., 2013). ACCg activity, on the other hand, is linked to shared experience and social reward, since neurons in the ACCg encoded reward allocations to another monkey, to oneself or to both (Chang et al., 2013). Such functional division between ACCs and ACC gyrus was also reported in imaging studies (Behrens et al., 2008; Apps and Ramnani, 2014). Consistent with these findings, lesions that include the rostral part of the monkey ACC induce deficits in social and emotional behaviors, such as a reduction in socially interactive behaviors and time spent in proximity with other individuals (Hadland et al., 2003). Interestingly, specific lesions to the ACCg decreased the social interest of monkeys to other individuals, while lesions to the ACCs did not (Rudebeck et al., 2006). Although further studies involving selective lesions within the ACC are required to investigate the functional differentiation within the rostro-caudal axis of the ACCg, these results corroborate the hypothesis that the rostral ACCg activity has a role in the engagement in spontaneous social interactions.

Human studies involving noninvasive imaging techniques have suggested that the medial prefrontal cortex, including the ACC, is involved in social cognition and social behaviors (Rushworth et al., 2007). The ACC, especially its rostral (pregenual) part, is activated during various social cognition tasks, including the prisoner's dilemma tasks, social judgments, and mentalizing (Rilling et al., 2002; Amodio and Frith, 2006; Mitchell et al., 2006; Tomlin et al., 2006). Furthermore, the activity of the medial prefrontal cortex, including the rostral ACC, increases in response to social gaze shifts compared to unsocial gaze shifts (Bristow et al., 2007). Accordingly, in humans, damage to the prefrontal cortex, including the ACC, induces changes in face expression identification and social behaviors and disturbs performance in a theory of mind task (Hornak et al., 2003; Baird et al., 2005).

Together, all these studies suggest that the rostral (pregenual) ACCg may play a specific role in social cognition. This hypothesis is corroborated by the present findings: the activity of neurons in the pregenual ACCg encoded specific social behaviors of the partner monkeys during spontaneous social interactions. Furthermore, the pregenual ACCg neurons did not respond to non-social behaviors, such as self-grooming, suggesting that the observed activity changes cannot be ascribed to non-specific arousal responses and that this area may have a role in engaging at social interactions. Previous neurophysiological studies have reported that neurons that are visually responsive to various non-social and emotional (rewarding and aversive) stimuli are located in the rostral part of the ACC in monkeys (Nishijo et al., 1997; Matsumoto et al., 2007). However, these neurons are mainly located in the ACCs (Nishijo et al., 1997; Vogt et al., 2005; Matsumoto et al., 2007), further suggesting a functional specialization between rostral ACCg and rostral ACCs.

## Conclusions

In this Hypothesis and Theory article we addressed the hypothesis that a sub-region of the ACC, the pregenual ACCg, has a central role in spontaneous social interactions. Our experimental findings, combined with previous reports in literature, support such hypothesis. Therefore, deficits in this region may be related to the pathology of the social deficits observed in psychiatric patients, such as those with schizophrenia and autism.

## Author contributions

HisN conceived and designed research; CM, JM, MA, and EH performed research; CM, JM, HirN, AT, and HisN analyzed data; CM, MA, HirN, TO, and HisN wrote and revised the paper.

### Conflict of interest statement

The authors declare that the research was conducted in the absence of any commercial or financial relationships that could be construed as a potential conflict of interest.
